# Noncentrosymmetric Lanthanide-Based MOF Materials Exhibiting Strong SHG Activity and NIR Luminescence of Er^3+^: Application in Nonlinear Optical Thermometry

**DOI:** 10.1021/acsami.2c22571

**Published:** 2023-01-05

**Authors:** Marcin Runowski, Dawid Marcinkowski, Kevin Soler-Carracedo, Adam Gorczyński, Ernest Ewert, Przemysław Woźny, Inocencio R. Martín

**Affiliations:** † Departamento de Física, Universidad de La Laguna, Apdo. Correos 456, E-38200San Cristóbal de La Laguna, Santa Cruz de Tenerife, Spain; ‡ Faculty of Chemistry, Adam Mickiewicz University, Uniwersytetu Poznańskiego 8, 61-614Poznań, Poland

**Keywords:** nonlinear optical thermometry via SHG, metal-organic frameworks, lanthanide MOFs, Er^3+^ NIR emission, second-harmonic generation, self-monitoring optical temperature sensor

## Abstract

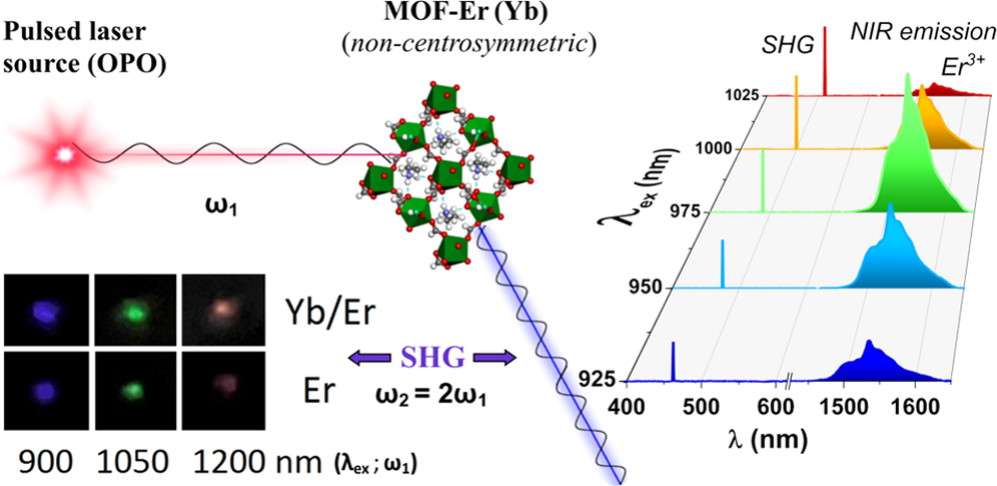

Optically active luminescent materials based on lanthanide ions attract significant attention due to their unique spectroscopic properties, nonlinear optical activity, and the possibility of application as contactless sensors. Lanthanide metal-organic frameworks (Ln-MOFs) that exhibit strong second-harmonic generation (SHG) and are optically active in the NIR region are unexpectedly underrepresented. Moreover, such Ln-MOFs require ligands that are chiral and/or need multistep synthetic procedures. Here, we show that the NIR pulsed laser irradiation of the noncentrosymmetric, isostructural Ln-MOF materials (MOF-Er^3+^ (1) and codoped MOF-Yb^3+^/Er^3+^ (2)) that are constructed from simple, achiral organic substrates in a one-step procedure results in strong and tunable SHG activity. The SHG signals could be easily collected, exciting the materials in a broad NIR spectral range, from ≈800 to 1500 nm, resulting in the intense color of emission, observed in the entire visible spectral region. Moreover, upon excitation in the range of ≈900 to 1025 nm, the materials also exhibit the NIR luminescence of Er^3+^ ions, centered at ≈1550 nm. The use of a 975 nm pulse excitation allows simultaneous observations of the conventional NIR emission of Er^3+^ and the SHG signal, altogether tuned by the composition of the Ln-MOF materials. Taking the benefits of different thermal responses of the mentioned effects, we have developed a nonlinear optical thermometer based on lanthanide-MOF materials. In this system, the SHG signal decreases with temperature, whereas the NIR emission band of Er^3+^ slightly broadens, allowing ratiometric (Er^3+^ NIR 1550 nm/SHG 488 nm) temperature monitoring. Our study provides a groundwork for the rational design of readily available and self-monitoring NLO-active Ln-MOFs with the desired optical and electronic properties.

## 1 Introduction

The development of smart, nano-, or micron-sized materials exhibiting diverse functionalities is a challenging task for researchers and engineers. Metal-organic frameworks (MOFs) attract increasing interest among such smart materials,^1−3^ mainly thanks to the combination of the organic and inorganic features in a single crystal structure, their structural flexibility, ease of modification, and multifunctionality.^4,5^ Some of the MOF materials may exhibit luminescence or magnetic properties, allowing for their application in optical sensing (e.g. luminescent thermometers),^6−10^ imaging techniques,^2,11,12^ or as single-molecular magnets,^13,14^ which arise through variations of metal ions, linkers, encapsulated guest, and/or surface functionalization.^1^ Thanks to their high surface-to-volume ratio and great porosity, MOFs can also be applied in catalysis,^15^ energy storage/conversion,^16^ separation techniques, as chemosensors, contrast agents for microscopy, and so forth.^17−22^

Given that most MOFs are centrosymmetric, because of the presence of an inversion center, the development of noncentrosymmetric MOFs has remained a significant challenge. These can be acquired through either the utilization of chiral ligands and/or templates, mostly with Zn(II)/Cd(II) ions as metallic nodes,^3^ or as recently demonstrated *via* the surface-coordinated MOF chemistry.^18,23^ Unexpectedly, the Ln-MOFs that exhibit nonlinear optical activity such as second-harmonic generation (SHG) are much less studied^17,24,25^ and are very scarce.^26,27^ This nonlinear optical process typically requires the use of coherent and collimated, excitation light source of high-energy density, such as a focused pulsed laser beam.^28^ The SHG phenomenon is an instantaneous and polarization-sensitive process, based on the energy conversion of the lower-energy fundamental beam, passing through the nonlinear optical medium, into the frequency-doubled second-harmonic beam.^29,30^ In other words, two low-frequency (ω) photons are converted into a single photon of high frequency (2ω). Importantly, the whole electromagnetic energy is conserved during the SHG process, and there is no real absorption of the incident light (fundamental beam) by the nonlinear optical medium, limiting the intrinsic, laser-induced heating of the system and the undesired photobleaching phenomena (SHG is governed by the virtual excited states).^28,31^ Hence, the SHG phenomena are commonly utilized in photonics, laser technology, microscopy imaging, sensing techniques, and so on.^17,18,28−30,32^

Nowadays, the vast majority of modern, optically active luminescent materials are based on lanthanide ions.^31,33−35^ This is mainly due to their (I) unique, ladder-like electronic structure, resulting in the abundance of emission lines in the UV, visible, and NIR spectral ranges; (II) characteristic and narrow absorption and emission bands, associated with shielding of the 4f electrons by the 5s and 5p ones; and (III) long emission lifetimes (μs–ms), originating from the forbidden intraconfigurational 4f–4f transitions. Moreover, some of the lanthanide ions embedded in the structure of the organic and/or inorganic (nano)materials may exhibit desired magnetic properties,^36−38^ selective catalytic activity,^39,40^ energy upconversion capability,^41−43^ as well as great pressure and/or temperature sensing performance.^33,43−46^ In fact, lanthanides are the main activator ions for most of the modern optical (contactless) thermometers, utilizing the effect of temperature-dependent luminescence for temperature monitoring (readouts) in a system of interest, including, e.g., optoelectronic devices, biological structures, as well as nanosized molecular magnets.^33,36−38,46,47^ Initially, only the lanthanide ions having thermally coupled levels—TCLs (*i.e*., two excited states separated by 200–2000 cm^–1^), such as Nd^3+^, Er^3+^, and Tm^3+^ ions, were used for luminescence thermometry, later called Boltzmann-type thermometers.^33,43,44,48,49^ However, in the last years, there has been an increasing amount of reports dealing with non-TCLs of lanthanides and the use of the corresponding, temperature-dependent band intensity ratios, emission line shifts, and even luminescence lifetimes as thermometric parameters (non-Boltzmann thermometers).^45,46,50−53^ One of the first papers about nonlinear temperature sensing was reported in 2010 by the group of J. García Solé, concerning the 2-photon fluorescence of CdSe quantum dots.^54^ Recently, there appeared first reports dealing with lanthanide-doped (Tm^3+^, Ho^3+^, or Er^3+^) BaTiO_3_ and NaNbO_3_ polycrystalline, inorganic materials, exhibiting SHG and upconversion luminescence properties, showing the possibility of their application in nonlinear optical thermometry.^28,31,55^ Rational design strategies for readily available NLO-active Ln-MOFs, especially those that are active in the NIR region, are, however, yet to be established, with the main bottleneck being the complex structure of used ligands and consequently the generation of high costs that hinder the practical applications.^1,11,17,56−58^

Here, we show the use of a noncentrosymmetric, lanthanide-based (Yb^3+^/Er^3+^) MOF for the nonlinear optical thermometry, constructed from simple, readily available achiral organic substrates. The synthesized MOF materials exhibit SHG activity in a whole visible spectral range and NIR emission of Er^3+^, centered around 1550 nm, upon pulsed laser excitation. Importantly, excitation of the materials in the range of ≈900 to 1025 nm allows simultaneous observations of the Er^3+^ emission and SHG signal, whose intensities are temperature-dependent. These features were further employed as thermometric parameters for the noninvasive temperature sensing. A facile, one-pot synthetic strategy from simple precursors and the modular character of synthesized systems render this class of materials very promising for further studies related to anticounterfeiting purposes, optical imaging, and self-monitoring of temperature.

## 2 Experimental Section

### 2.1 Synthesis

#### 2.1.1 Preparation of Compounds (**1**) and (**2**)

The metal salts and solvents were supplied by Sigma-Aldrich and POCH. All chemicals mentioned above were of analytical-grade quality and were used as obtained without further purification. Compounds (**1**) {[EtNH_3_]Er(HCOO)_4_} and (**2**) {[EtNH_3_]Yb_0.79_Er_0.21_(HCOO)_4_} were synthesized with a modified procedure.^59^ In a 100 mL pressure Hastelloy C-22 steel reactor EasyMax (Mettler Toledo), an appropriate lanthanide(III) salt (**1**) of 1.77 g (4 mmol) Er(NO_3_)_3_ and (**2**) of 0.35 g (0.8 mmol) Er(NO_3_)_3_ and 1.44 g (3.2 mmol) Yb(NO_3_)_3_ were placed and dissolved in a mixture of 25 mL of *N*-ethylformamide (Sigma-Aldrich), 15 mL of MilliQ water, and 5 mL of 98 wt % HCOOH. Then, the reactor was closed and filled with argon to increase an internal pressure up to 20 bar. The reaction mixture was stirred (800 rpm.) and heated to 140 °C (rate 5 °C/min.) for 24 h. After this time, the reaction was slowly cooled to 1 °C (rate 0.1 °C/min.). The pressure in the reactor was equalized. The dark reaction mixture was filtrated and left to slowly evaporate in a Petri dish under a fume hood. First, pink (**1**)/pinkish (**2**) crystals were visible after 4 days, but the final products in a high yield were obtained after 2 weeks. Crystals were washed with ethanol (3 × 15 mL) and dried in a BUCHI Glass Oven at 40 °C under reduced pressure for 24 h. The final composition of (**2**) was determined as [CH_3_CH_2_NH_3_]Yb_0.79_Er_0.21_(HCOO)_4_ on the basis of the ICP-OES analysis. The structural characterization details are given in the Supporting Information (SI) data.

### 2.2 Characterization

Fourier transform near-infrared (FT-IR) spectra were obtained with a Bruker IFS 66v/S spectrophotometer, and peak positions are reported in cm^–1^. Powder X-ray diffraction (PXRD) analyses were performed using a Bruker AXS D8 Advance diffractometer. A powdered microcrystalline sample was ground in an agate mortar and deposited in the hollow of a quartz zero-background plate. ICP-OES analysis was performed with an ICP-OES Vista-MPX in accordance with the Polish Standard PN-EN-ISO-11885_2009E. Thermogravimetry (TG) analysis was conducted for crystalline samples of compounds on a Setsys 1200 Setaram apparatus. Samples were placed in an open corundum crucible and measured with a heating rate of 5 °C/min. The measurements were performed in a temperature range of 10–1000 °C under a He atmosphere. Elemental analysis was performed using a PerkinElmer 2400 CHN microanalyser on fully desolvated samples (drying for 24 h under vacuum at 40 °C). A tunable pulsed laser EKSPLA/NT342/3/UVE 10 ns/10 Hz optical parametric oscillator (OPO) was used as an excitation source. For all experiments, the energy of the laser pulse was adjusted to ≈1 mJ and the spot size was ≈1 mm (power density of approx. 1 W/cm^2^). The emission spectra, *i.e*., SHG signals and NIR emission of Er^3+^, were acquired by the use of an Andor Shamrock 500 spectrometer coupled to the silicon and InGaAs CCD cameras, respectively, and they were corrected for the apparatus response. The spectroscopic measurements of luminescence and SHG signals under high-temperature conditions were performed in a tubular electric furnace (Gero RES-E 230/3), having a type K thermocouple in contact with the sample, to precisely monitor its temperature.

## 3 Results and Discussion

### 3.1 Structural Properties

**Figure 1:**
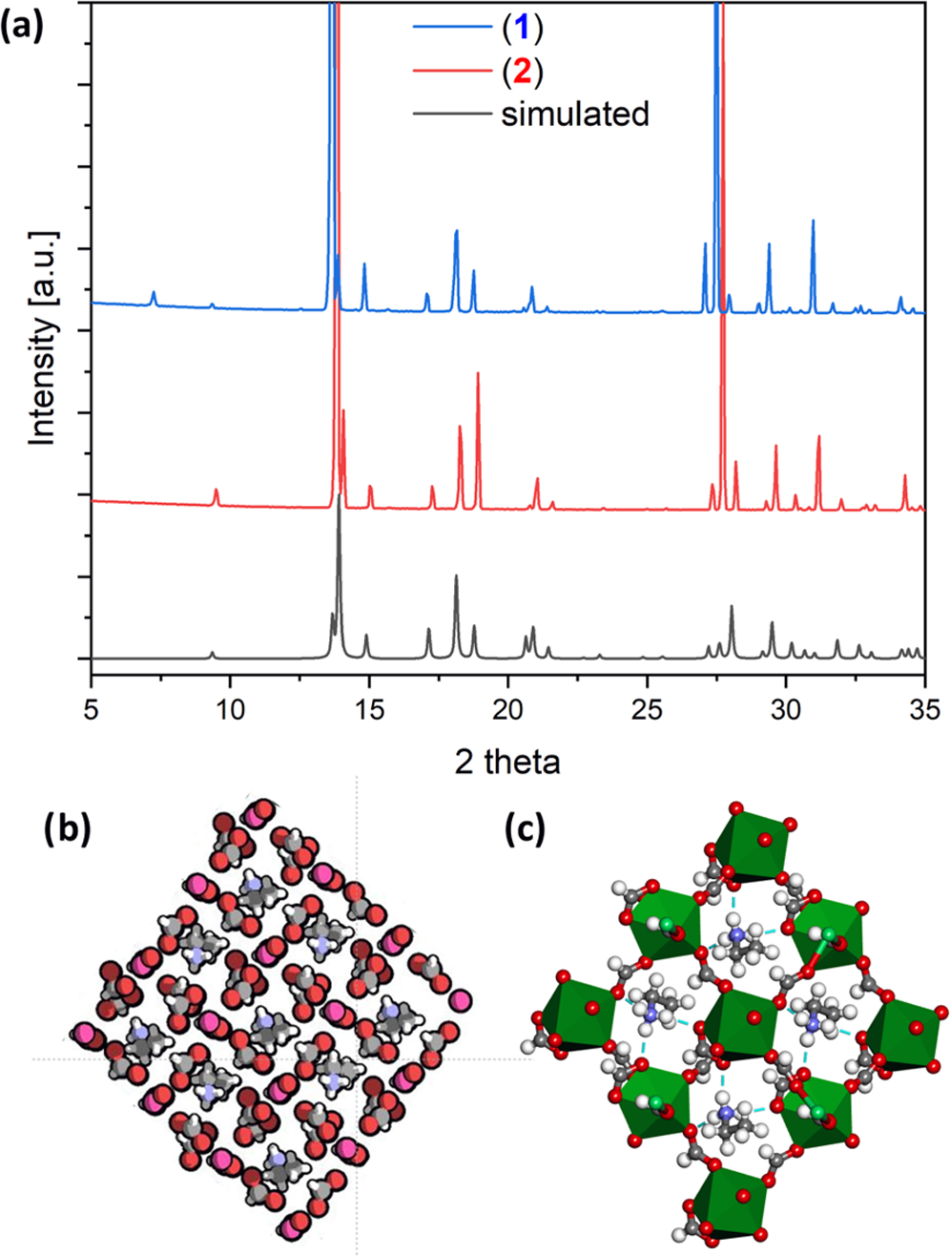
(a) Experimental and simulated PXRD spectra for synthesized compounds, i.e., MOF-Er and MOF-Yb/Er. (b) Crystal structure of the MOF-Ln (Ln = Er^3+^ or Yb^3+^/Er^3+^) as seen along the a-direction. (c) Polyhedral representation of the MOF-Ln crystal structure showing the N–H···O hydrogen bonds (blue dashed lines) between the protonated amine and formate framework. Color key: Er^3+^/Yb^3+^, green; oxygen, red; nitrogen, blue; carbon, gray; hydrogen, white; and [Ln^3+^O_8_], green polyhedra. Hydrogen atoms are shown as spheres of arbitrary radii.

The structure and uniformity of the samples were checked with PXRD analysis and compared with the deposited structure (1556097) in Cambridge Crystallographic Data Centre. The experimental and simulated spectra are demonstrated in Figure 1a, which confirm excellent compliance with the simulated spectra for both compounds and further confirm their isostructural character. Ln^3+^ cations are coordinated by bidentate HCOO^–^ formate anions to form Ln-MOF networks {[EtNH_3_]Er(HCOO)_4_} (**1**) and {[EtNH_3_]Yb_0.79_Er_0.21_(HCOO)_4_} (**2**) that crystallize in a polar and noncentrosymmetric monoclinic system (*P*2_1_ space group). The graphical representation of the crystal structure of the Ln-MOF (Ln = Er or Yb/Er) seen along the *a*-direction is given in Figure 1b,c, including its polyhedral representation showing the N–H···O hydrogen bonds between the protonated amine and the formate framework. Interestingly, the Ln-MOF formate scaffold can be modulated to a certain degree with the structure of the protonated amine, and the noncentrosymmetric character of the MOF is retained.^60^ Characterization was additionally supported by FT-IR data (Figure S1), elemental analysis, and DTA studies (Figure S2), which are given in the SI file. Importantly, for both complexes, the DTA analyses revealed good thermal stability (up to 180 °C), which is a prerequisite for high-temperature sensor studies. Please note that in contrast to the known examples of Ln-MOFs that exhibit SHG properties, they necessitate ligands acquired through multistep synthetic procedures,^17,24,25^ whereas the Ln-MOFs synthesized herein are obtained in a one-pot procedure from simple, achiral and readily available substrates (see also Schemes 1 and 2 in the SI).

### 3.2 Optical Activity

**Figure 2:**
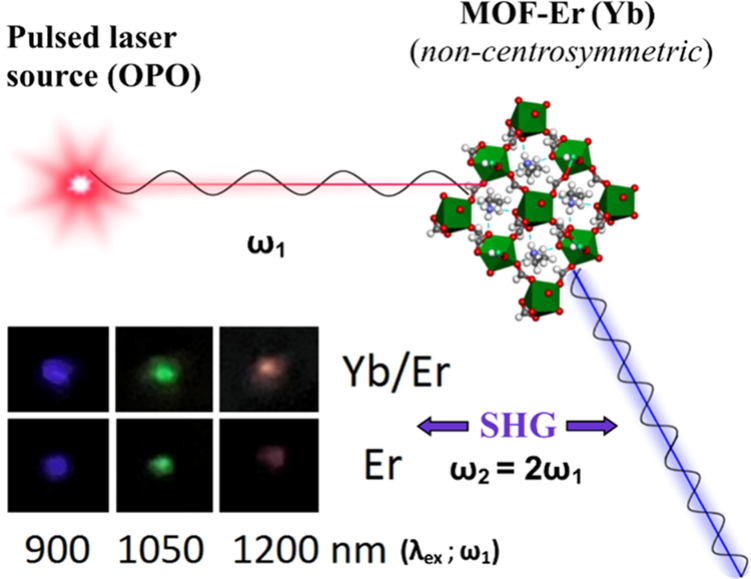
Schematic representation of the SHG phenomenon in the synthesized noncentrosymmetric, lanthanide-based MOFs; the inset (left-bottom) shows the digital images of the MOF-Yb^3+^/Er^3+^ (top row) and MOF-Er^3+^ (bottom row) samples taken under pulsed laser irradiation (λ_ex_ = 900, 1050, and 1200 nm), showing the bright colors of the SHG light observed in a visible range.

To check the nonlinear activity of the synthesized MOF materials, we have directly irradiated the polycrystalline samples with a focused beam of the frequency-tunable (ω_1_), nanosecond pulsed laser (OPO), as schematically presented in Figure 2. Both MOFs exhibited a bright SHG signal (ω_2_ = 2ω_1_), clearly visible by the naked eye, namely, the blue color (450 nm) for the λ_ex_ = 900 nm, green (525 nm) for the λ_ex_ = 1050 nm, and red (600 nm) for the λ_ex_ = 1200 nm (see the inset in the left-bottom corner of Figure 2). As expected, the SHG signal was stronger (brighter colors) for the MOF containing less Er^3+^ ions, *i.e*., the sample labeled as “Yb/Er” (**2**), which contains 79 mol % of Yb^3+^ and 21 mol % of Er^3+^ (top row). This is mainly due to the strong absorption of Er^3+^ in the visible range, resulting in a partial reabsorption of the emitted SHG signal.^61^ Moreover, the codoped sample containing both lanthanide ions exhibited higher NIR luminescence intensity of Er^3+^, centered around 1550 nm (see Figure S3). This is due to the large absorption cross section of Yb^3+^, resulting in the efficient resonant energy transfer from Yb^3+^ to Er^3+^ ions, which enhances Er^3+^ emission.^51,52^

**Figure 3:**
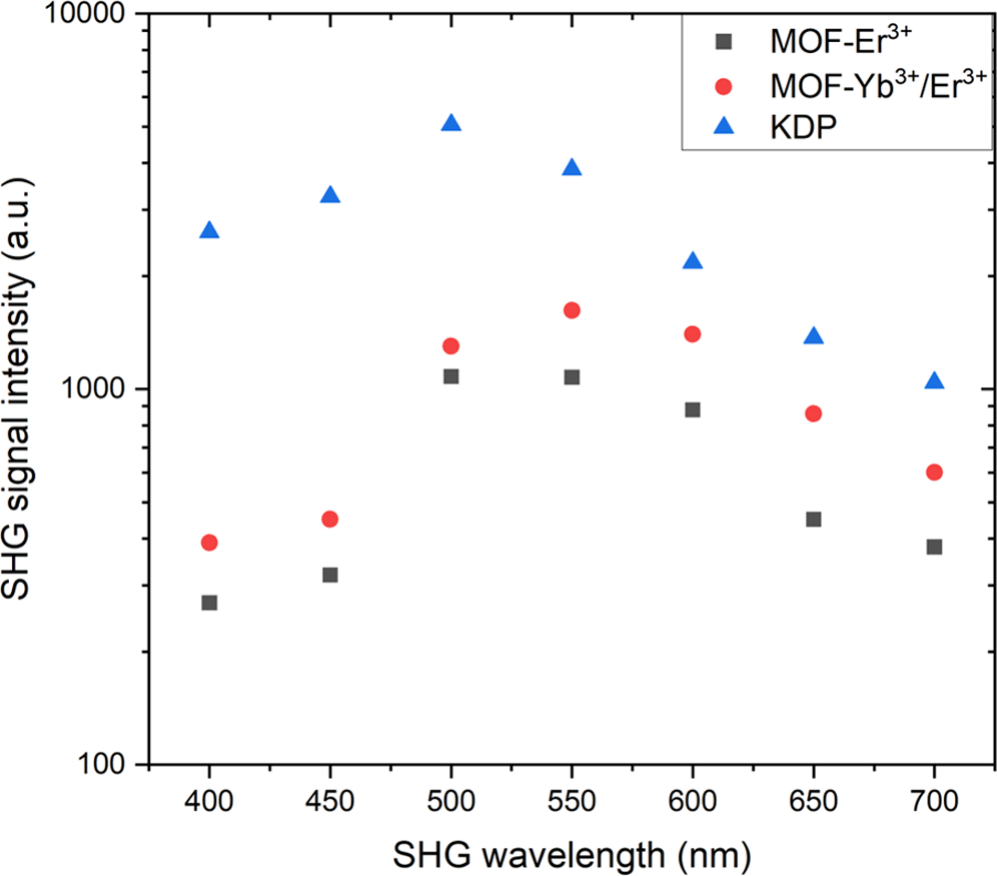
Comparison of the SHG signal intensities for the polycrystalline MOF-Er^3+^ and MOF-Yb^3+^/Er^3+^ materials with KDP (reference), as a function of the excitation wavelength (λ_ex_ = 800–1400 nm); the crystal sizes for all compounds were fixed to ≈30 to 50 μm.

To quantitatively compare the performance of the SHG of the synthesized lanthanide-based MOF materials, we have used the commercially available KDP crystals as a reference and measured the SHG signal at different excitation wavelengths. To ensure a reliable intensity comparison, before the measurements all compounds were gently ground into powders to obtain crystal sizes of ≈30 to 50 μm. It is worth noting that the mentioned procedure is a common and simple way for quantitative comparisons of the SHG signal intensity for powder, polycrystalline, ceramic, or layer-type materials, successfully used elsewhere by others.^17,62−64^Figure 3 shows the mentioned comparison of the SHG intensity as a function of excitation wavelength (λ_ex_ = 800–1400 nm). Generally, the SHG intensities for the reference KDP crystals are higher than for the lanthanide-based MOF materials; nonetheless, for the λ_ex_ = 1200–1400 nm, the signal intensities for the synthesized MOFs are only slightly lower compared to the KDP. However, it should be kept in mind that the inorganic KDP crystals are commercially used as a very efficient material for the generation of second harmonics, so the obtained SHG intensities from the organic compounds are quite good, especially given how rare the noncentrosymmetric emissive Ln-MOFs are. Hence, the MOFs studied are promising candidates as modern, organic-based nonlinear optical materials, noting very simple, achiral organic precursors and a high-yielding synthetic method. Finally, it is worth noting that for all λ_ex_ used, the SHG intensities originating from the MOF-Yb^3+^/Er^3+^ material (**2**) are higher than for the MOF-Er^3+^ (**1**) one. As already mentioned, the lower signal intensities of the Er^3+^-based MOF are most plausibly the result of a higher content of Er^3+^ in that compound, leading to stronger reabsorption of the fundamental and second-harmonic beams.^61^

**Figure 4:**
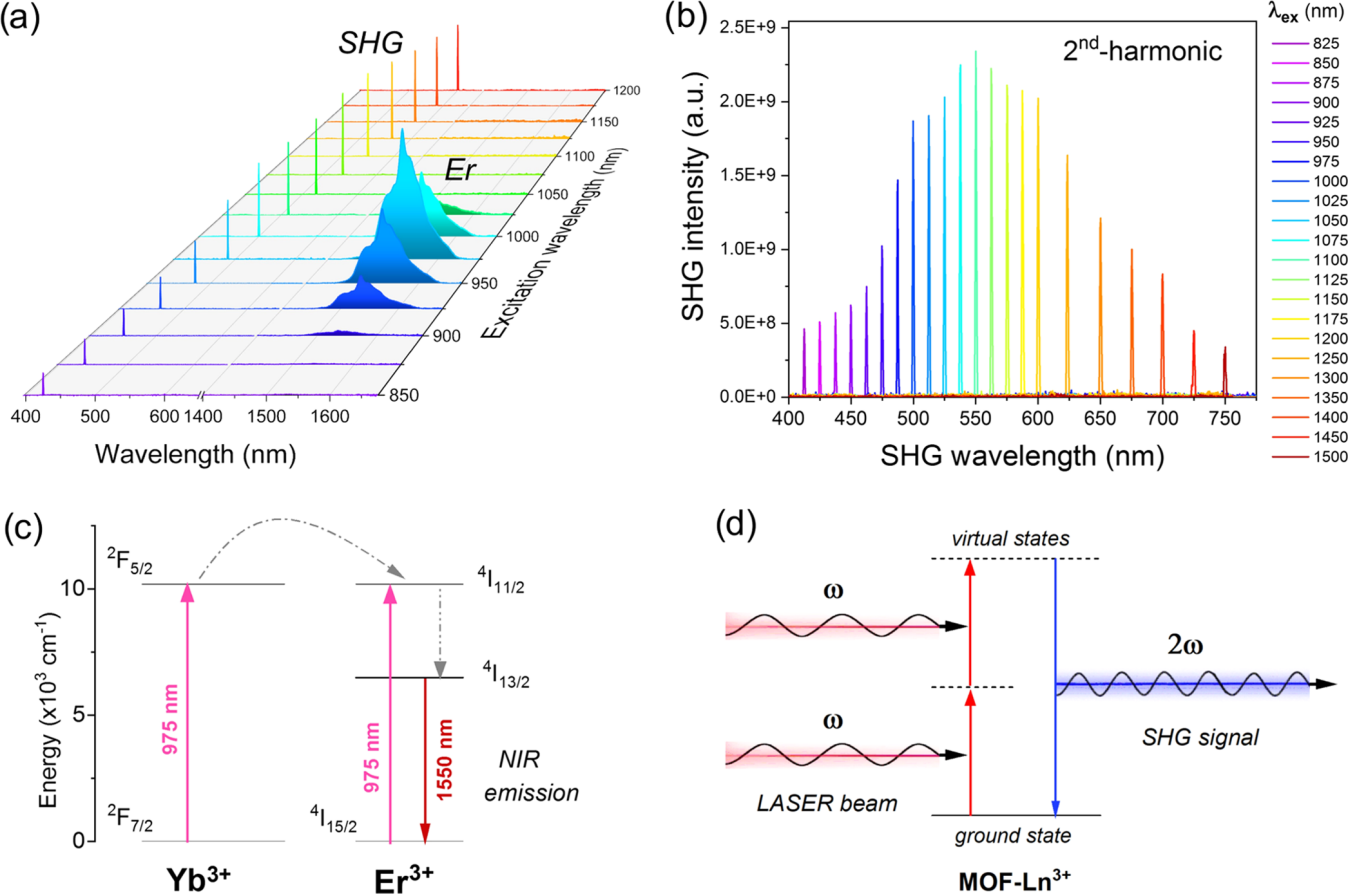
(a) Selected emission spectra for the MOF-Yb^3+^/Er^3+^ material (**2**), showing SHG and NIR luminescence of Er^3+^ (centered around 1550 nm), recorded at different excitation wavelengths (λ_ex_ = 850–1200 nm); (b) overlaid SHG spectra obtained for the same compound, presenting the SHG signal acquired in a broader excitation range (λ_ex_ = 825–1500 nm); (c) energy level diagram depicting the discussed radiative and nonradiative processes between Yb^3+^-Er^3+^ ions and emphasizing the NIR luminescence of Er^3+^; and (d) energy level diagram explaining the SHG mechanism in the investigated lanthanide-based MOF materials.

Due to the above-mentioned effects, *i.e*., stronger Er^3+^ NIR emission and better SHG signal intensity, we have selected the Yb^3+^/Er^3+^-based MOF (**2**) for further experiments. We have investigated the optical activity (*i.e*., Er^3+^ NIR emission and SHG) of this material in the broader spectral range, ranging from ≈400 to 1700 nm (Figure 4a), recording the emission spectra at each excitation wavelength, with an increment of 25 nm for the λ_ex_ = 825–1200 nm and every 50 nm for longer wavelengths (Figure 4b). Exciting the sample in the spectral range from ≈900 to 1025 nm, the material studied exhibits simultaneously alike SHG signal and NIR emission of Er^3+^. This is due to the broad spectral absorption of Yb^3+^ in that range, whose absorption band is centered around 975–980 nm, corresponding to the ^2^F_7/2_ → ^2^F_5/2_ transition.^61^ Hence, the excitation energy can be further effectively transferred to the emitting Er^3+^ ions, *via* Yb^3+^ → Er^3+^ (^2^F_5/2_ → ^4^I_11/2_) energy transfer. Subsequently, due to the nonradiative, multiphonon relaxation process, the excited electron moves to the lowest excited state of Er^3+^ (^4^I_11/2_ → ^4^I_13/2_). Finally, the electron relaxes radiatively from the emitting level to the ground state of Er^3+^ (^4^I_13/2_ → ^4^I_15/2_), accompanied by the NIR luminescence, manifested as a broad band centered around 1550 nm, which is present in the emission spectra recorded at λ_ex_ ≈ 900–1025 nm (see Figure 4a). As expected, the greatest intensity of Er^3+^ NIR emission occurs at λ_ex_ = 975 nm, being consistent with the maximum absorption of Yb^3+^, whereas, the highest intensities of the SHG lines are observed in the middle of the visible spectral range, *i.e*., around ≈500–600 nm (λ_ex_ = 1000–1200 nm). Please note that in the case of the MOF-Er^3+^ compound **1** (without Yb^3+^), the NIR emission of Er^3+^ is a result of the ground-state absorption process (Er^3+^: ^4^I_15/2_ → ^4^I_11/2_), *i.e*., occurs *via* a less efficient, direct excitation of Er^3+^ ions. The corresponding energy level diagrams, graphically showing the mentioned radiative and nonradiative processes between the lanthanide ions, and the SHG mechanism are presented in Figure 4c,d, respectively.

### 3.3 Temperature-Dependent Measurements

**Figure 5:**
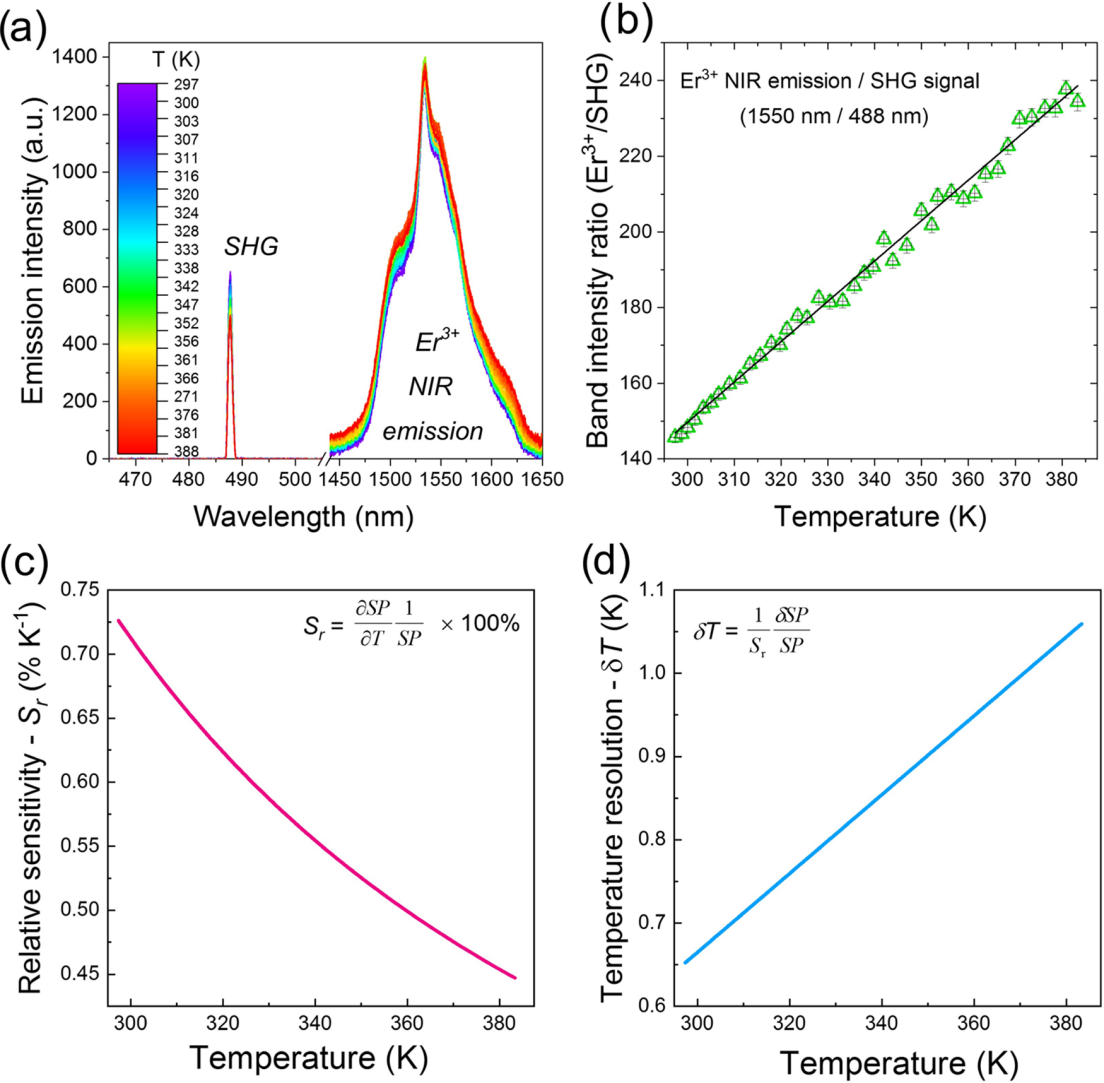
(a) Emission spectra for the synthesized MOF-Yb^3+^/Er^3+^ (**2**), recorded at different temperature values, at a pulsed laser excitation of 975 nm, showing the SHG signal and NIR emission of Er^3+^; (b) determined band intensity ratios—Er^3+^/SHG (green triangles) and the applied linear fit (black solid line); and (c) the corresponding *S*_r_ and (d) δ*T* values as a function of temperature.

In the final step, we investigated the optical activity of the synthesized MOF-Yb^3+^/Er^3+^ material (**2**) as a function of temperature, showing its potential as a new, nonlinear optical thermometer. We have measured the emission spectra for this MOF compound in the *T*-range from 297 to 388 K, monitoring both the SHG signal in the visible range and the emission of Er^3+^ in the NIR region (Figure 5a). It is clearly seen that the SHG signal decreases with temperature, whereas the emission band of Er^3+^ broadens; hence, its integrated intensity slightly increases with temperature elevation. The observed thermal broadening effects of Er^3+^ NIR emission are associated with enhanced electron–phonon coupling at elevated temperature (vibrational contribution) and slight deviations from the initial geometry of the Ln^3+^ site in the crystals due to the lattice thermal expansion effects (static contribution), whereas the decrease of the SHG signal intensity with temperature is plausibly associated with increasing strains and distortions in the crystals, as well as enhanced absorption of Er^3+^ ions at elevated temperature, leading to the greater reabsorption of the SHG light.^55^ DTA analysis excluded the possibility of structural interconversions in the studied *T*-range related to the loss of solvent molecules and eventual structural collapse of the MOF framework (Figure S2). Thanks to the opposite behaviors of Er^3+^ NIR luminescence and SHG with temperature, we could calculate the corresponding band intensity ratios (*I*_Er_/*I*_SHG_) as a function of temperature, using the integrated intensities of both bands, and plot the resulting *I*_Er_/*I*_SHG_ values in Figure 5b. The calculated *I*_Er_/*I*_SHG_ ratio increases linearly with temperature in the investigated *T*-range, so a simple linear fit was applied to correlate the measured quantities, namely, *I*_Er_/*I*_SHG_ = 1.067*T* – 170.3, with *R*^2^ = 0.99.

To quantitatively analyze the sensing performance of each optical thermometer, it is necessary to determine its relative temperature sensitivities, *S*_r_ (%K^–1^), and temperature resolution (δ*T*). The *S*_r_ value shows how the determined spectroscopic parameter (SP) changes per 1 degree of the absolute temperature, and it is usually expressed as
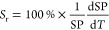
1whereas δ*T* is an uncertainty of temperature determination, typically estimated based on the following formula
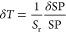
2where δSP is the uncertainty of the determined SP value, which is associated with a measured signal intensity *vs* a background level. Figure 5c,d presents how the *S*_r_ and δ*T* change as a function of temperature, namely, the *S*_r_ values decrease from ≈0.73 to ≈0.44%K^–1^, whereas the δ*T* values increase from ≈0.65 to 1.05 K by elevating the temperature from 297 to 388 K, respectively. The obtained results suggest that the studied MOF-Yb^3+^/Er^3+^ (**2**) can be used for optical thermometry applications.

To examine the reliability and repeatability of the proposed sensing method, we performed a series of cycling measurements, measuring the Er^3+^ NIR emission and SHG signal between the low and high temperatures. The resulting band intensity ratios are presented in Figure S4. It is clear that the SP values are determined and so the band intensity ratios change reversibly with temperature, confirming the validity of the proposed temperature sensing strategy.

Moreover, we have compared in Table S1 the performance of the developed nonlinear temperature sensor with other optical thermometers operating within a similar *T*-range, for which the temperature resolution, i.e., δ*T* data, is available. To provide a reliable comparison, we provided the values of sensitivity and resolution at a fixed temperature, *i.e*., 313 K, which is located in the physiological range and is important from the biological (optical temperature detection for hyperthermia and tumor monitoring) and industrial points of view (temperature monitoring of electronics). It is clear that despite relatively low *S*_r_ values, compared to other optical thermometers, the investigated MOF-Yb^3+^/Er^3+^ material exhibits very good thermal resolution, i.e., δ*T* ≈ 0.7 K at 313 K, which in fact is a crucial parameter for all optical temperature sensor materials.

## 4 Conclusions

Here, we have shown the possibility of utilizing the lanthanide-based MOF materials for the nonlinear optical thermometry based on the SHG phenomena and NIR luminescence of Er^3+^ ions. Synthesized MOFs are constructed in a one-pot, high-yielding procedure from simple, readily available achiral organic precursors and retain the modular character, so that isostructural pure MOF-Er^3+^ (**1**) and codoped MOF-Yb^3+^/Er^3+^ (**2**) can be obtained. Thanks to the lack of an inversion center, the synthesized materials are noncentrosymmetric (in contrast to most MOFs); hence, they can exhibit nonlinear optical activity. We have used this favorable feature to generate second-harmonic electromagnetic waves in the whole visible spectral range upon nanosecond pulsed laser excitation. Importantly, by exciting the investigated MOFs around 900–1025 nm, it is possible to simultaneously generate the NIR emission of Er^3+^ ions (centered at ≈1550 nm), with efficiency amenable to the presence or absence of doping with Yb^3+^ ions. Hence, these materials may exhibit alike conventional photoluminescence properties (*i.e*., one-photon emission) and nonlinear optical activity, manifested as SHG, which is a second-order, two-photon process. Thanks to the different temperature dependences of these processes, we have developed a novel nonlinear optical thermometer based on the MOF-Yb^3+^/Er^3+^ material (**2**). An appropriate combination of both optical phenomena, *i.e*., the use of band intensity ratios of Er^3+^ emission to SHG signal (*I*_Er_/*I*_SHG_) as a thermometric parameter, allows temperature readouts with satisfactory sensitivity and resolution (<1 K). The developed sensor allows self-monitoring of the organic framework temperature, as well as it can be used for remote temperature monitoring of various systems, *i.e*., thermal characteristics of their surface and interior of the system (after appropriate calibration). This work shows new guidelines and perspectives for the lanthanide-based MOF materials, which are very simple to construct, allowing self-monitoring of their temperature, and their applications as active components of modern optoelectronic devices for optical sensing purposes, anticounterfeiting, energy conversion, optical imaging/mapping techniques, and so forth.

## Supporting Information Available

FT-IR data; elemental analysis results; synthetic schemes for known ligands that induce SHG phenomenon; DTA curves; NIR emission spectra; thermal cycling; and performance comparison of optical thermometers (PDF)
