# Supplementary material for: Noncentrosymmetric Lanthanide-Based MOF Materials Exhibiting Strong SHG Activity and NIR Luminescence of Er3+: Application in Nonlinear Optical Thermometry
Source: ACS Appl Mater Interfaces. 2023 Jan 5;15(2):3244–52. doi: 10.1021/acsami.2c22571 (PMC9869334; doi:10.1021/acsami.2c22571)
Supplement: Supplementary file 1 — am2c22571_si_001.pdf [file am2c22571_si_001.pdf]

Suporting Information for:

Non-centrosymmetric lanthanide-based MOF materials exhibiting strong SHG activity and NIR luminescence of Er^{3+} – application in non-linear optical thermometry

Marcin Runowski[1,2,*], Dawid Marcinkowski[2], Kevin Soler-Carracedo[1], Adam Gorczyński[*2], Ernest Ewert[2], Przemysław Woźny,[2] and Inocencio R. Martín[1]

[1]*Universidad de La Laguna, Departamento de Física, Apdo. Correos 456 E-38200, San Cristóbal de La Laguna, Santa Cruz de Tenerife, Spain*

[2]*Adam Mickiewicz University, Faculty of Chemistry, Uniwersytetu Poznańskiego 8, 61-614 Poznań, Poland*

Corresponding Authors

*Marcin Runowski: E-mail: mrunowsk@ull.edu.es or runowski@amu.edu.pl

*Adam Gorczyński: E-mail: adam.gorczynski@amu.edu.pl

Elemental analysis and IR data

{[$CH_3CH_2NH_3$]Er($HCOO)_4$} (**1**): Yield: 1.48 g, 95%. Elemental analysis calc. for [$C_6H_{12}ErNO_8$] (391.99): C, 18.32; H, 3.07; N, 3.56; found: C, 18.55; H, 3.24; N, 3.69. Selected IR (KBr, cm^{-1}): ν(N-H) 3426; ν_{as}(CH_3 and CH_2) 3074, 3047, 3019, 3001, 2978, 2947, 2923; ν_s(CH_3 and CH_2) 2865, 2829; δ_{as}(NH_3 and C=O) 1659, 1630; δ_s(C=O) 1596, 1577; δ_s(N-H) 1526; δ(CH_3 and CH_2) 1458; δ(CH_3) 1352; γ(CH_3) 1212; ν_{as}(CCN) 1052; γ(NH_3)1002; ν_s(CCN) 877; δ(Ln^{3+}- O) 425.

{[$CH_3CH_2NH_3$]$Yb_{0.79}Er_{0.21}$($HCOO)_4$} (**2**): Yield: 1.37 g, 88%. Elemental analysis calc. for [$C_6H_{12}Er_{0.21}NO_8Yb_{0.79}$] (398.39): C, 18.11; H, 3.04; N, 3.52; found: C, 18.11; H, 3.14; N, 3.51.

Selected IR (KBr, cm^{-1}): ν(N-H) 3427; ν_{as}(CH_3 and CH_2) 3074, 3047, 3019, 3004, 2979, 2947, 2920; ν_s(CH_3 and CH_2) 2865, 2823; δ_{as}(NH_3 and C=O) 1659, 1629; δ_s(C=O) 1599, 1577; δ_s(N-H) 1531; δ(CH_3 and CH_2) 1456; δ(CH_3) 1349; γ(CH_3) 1210; ν_{as}(CCN) 1050; γ(NH_3)1002; ν_s(CCN) 1051, 877; δ(Ln^{3+}- O) 425.

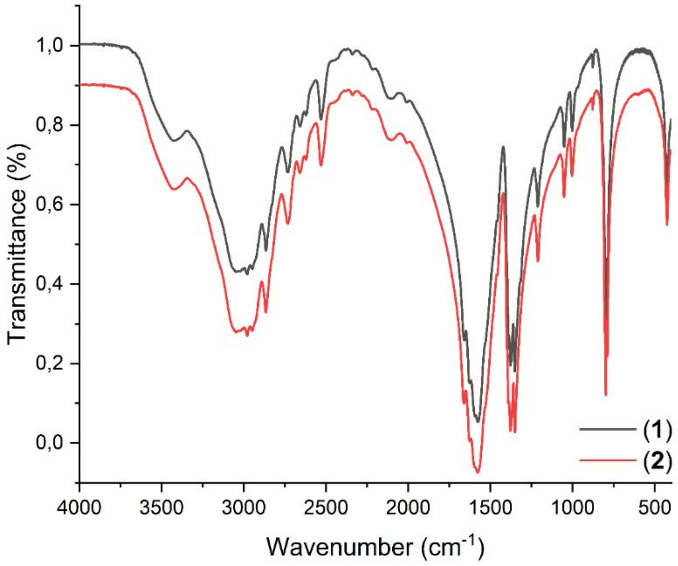

Figure S1. FT-IR spectra for compound (**1**) and (**2**), *i.e.*, MOF-Yb^{3+}/Er^{3+} and MOF-/Er^{3+} materials, respectively.

Thermogravimetry analysis

The TG curves indicate that a two-step decomposition process of this compounds occurs (see **Figure S2**). Firstly, between 150 - 200°C the decomposition of formate moieties (mass loss ~ 24%, 2 molecules of $CHOO^-$) what is accompanied by an endothermic peak on HF curve (HF_{max} = 186°C). Then, between 265 - 355°C the decomposition of formate moieties continues (mass loss ~ 47%, next 2 molecules of $CHOO^-$) endothermic peak at HF_{max} ~315°C. From this point a gradual degradation of the organic residues was observed. No constant mass is obtained up to 1000°C.

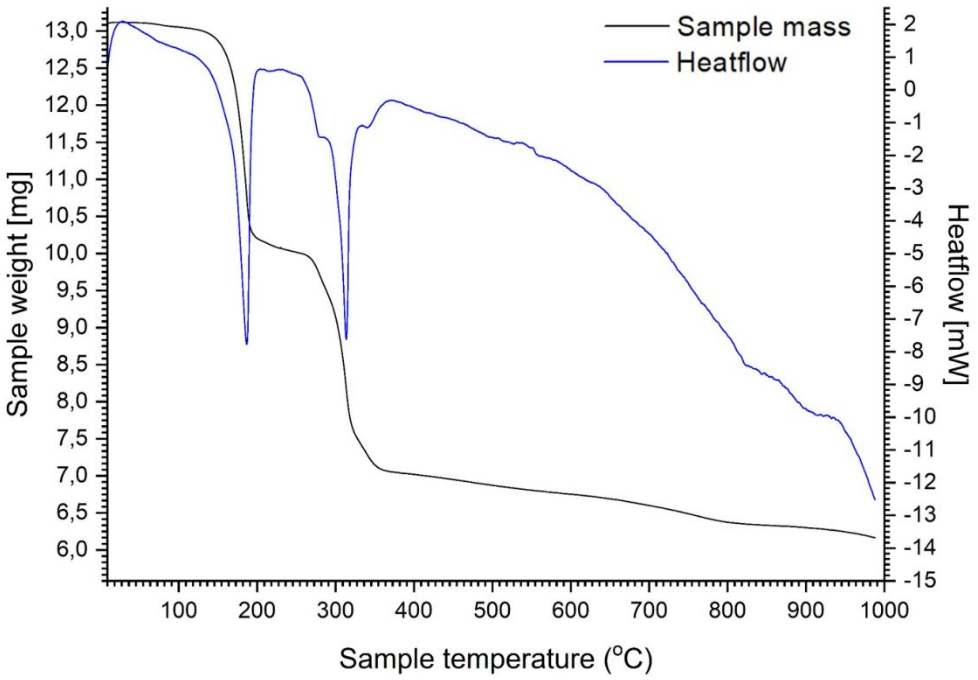

Figure S2. DTA analysis for compounds (**1**) and (**2**), *i.e.*, MOF-Yb^{3+}/Er^{3+} and MOF-/Er^{3+} materials, respectively.

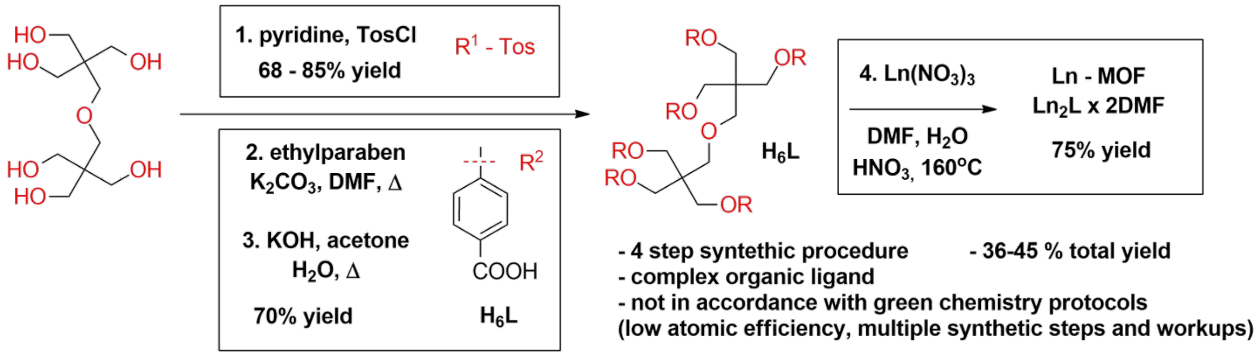

Scheme S1. Synthetic procedure towards H_6**L** ligand forms SHG active Ln-MOFs. Based on: Chem. Commun., 2012, 48, 11139–11141 and Inorg. Chem., 2021, 60, 7345−7350

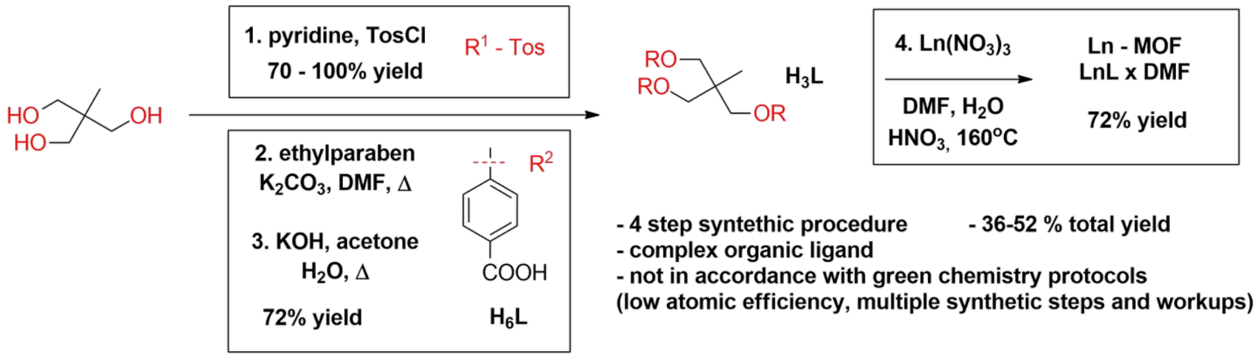

Scheme S2. Synthetic procedure towards H_3**L** ligand forms SHG active Ln-MOFs. Based on: Chem. Eur. J., 2013, 19, 17172–17179

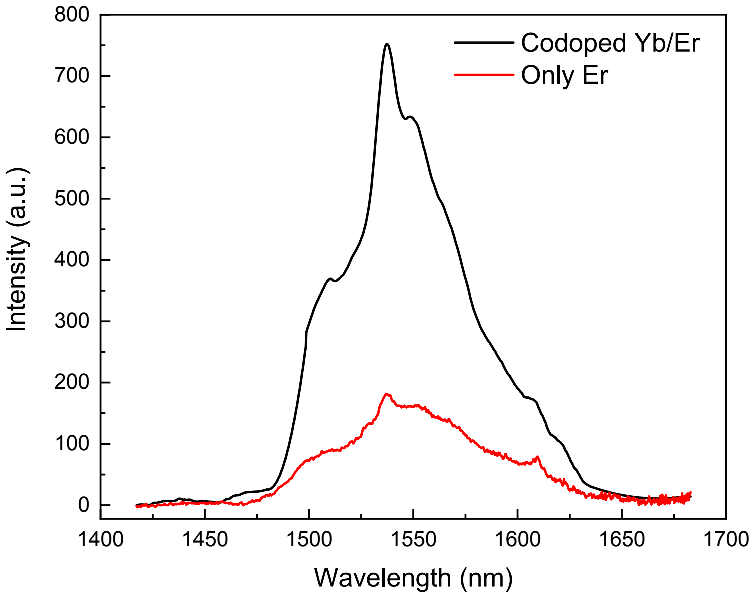

Figure S3. Emission spectra for the synthesized MOF-/Er^{3+} (**1**) and MOF-Yb^{3+}/Er^{3+} (**2**) materials, showing NIR emission of Er^{3+} (transition); λ_{ex} = 975 nm (pulsed excitation).

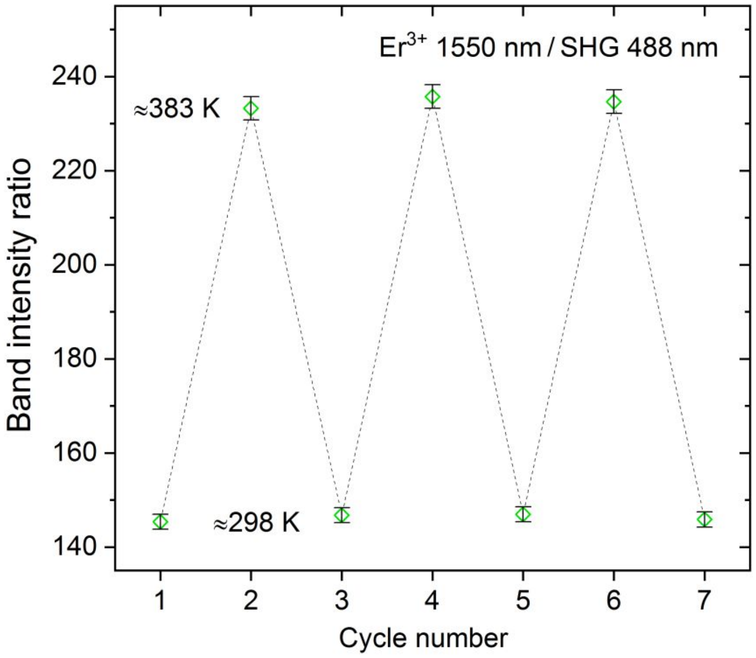

Figure S4. Thermal cycling, i.e. measurements of the band intensity ratio parameter (Er^{3+} NIR emission at 1550 nm / SHG signal at 488 nm), by cycling the sample between room and high temperature; λ_{ex} = 975 nm (pulsed excitation).

Table S1 Relative thermal sensitivities (S_r) and temperature resolutions (δT) at 40°C, for different lanthanide-based luminescent thermometers.

Material	Activator ions	S_r at 40°C (% K^{-1})	δT at 40°C (^{o}C)	T-range (^{o}C)	Transitions	λ (nm)	Ref.
MOF	Yb^{3+}, Er^{3+}	0.65	0.7	20-110	(Er^{3+}) $^4I_{13/2}$→$^4I_{15/2}$/ SHG signal	1550/488	This work
$BaTiO_3$	Yb^{3+}, Ho^{3+}	0.45	2.0	25–305	SHG signal / (Ho^{3+}) $^5S_2$, $^5F_4$→$^5I_8$	488/550	1
$BaTiO_3$	Yb^{3+}, Er^{3+}	0.55	0.6	23-300	SHG signal/ (Er^{3+}) $^2H_{11/2}$→$^4I_{15/2}$/	488/525	2
Sr_2LuF_7	Yb^{3+}, Ho^{3+}, Er^{3+}	4.35	0.6	20-56	(Yb^{3+}) $^2F_{5/2}$→$^2F_{7/2}$/ (Er^{3+}) $^2H_{9/2}$→$^4I_{15/2}$	950/410	3
		3.14	0.6		(Yb^{3+}) $^2F_{5/2}$→$^2F_{7/2}$/ (Ho^{3+}) $^3K_8$,$^5F_2$→$^5I_8$	950/490	
		1.25	0.8		(Er^{3+}) $^4I_{13/2}$→$^4I_{15/2}$/ (Yb^{3+}) $^2F_{5/2}$→$^2F_{7/2}$	1550/1000	
LaF_3	Pr^{3+}	0.55	1.0	−153-47	$^3P_1$→$^3H_5$/$^3P_0$→$^3H_5$	525/540	4
LaF_3:Nd /LaF_3 *core/shell*	Nd^{3+}	≈0.14 cm^{-1}/K	2.0	30-80	$^4F_{3/2}$→$^4I_{9/2}$ (Stark) (band shift)	863	5
β-$NaYF_4$/SiO_2 core/shell nanorods	Yb^{3+}, Er^{3+}	1.19	0.6	26-64	(Er^{3+}) $^2H_{11/2}$→$^4I_{15/2}$/ (Er^{3+}) $^4S_{3/2}$→$^4I_{15/2}$	525/545	6
		1.44	0.8		(Yb^{3+}) $^2F_{5/2}$→$^2F_{7/2}$/ (Er^{3+}) $^4I_{9/2}$→$^4I_{15/2}$	1010/810	
		0.79	1.0		(Yb^{3+}) $^2F_{5/2}$→$^2F_{7/2}$/ (Er^{3+}) $^4F_{9/2}$→$^4I_{15/2}$	1010/660	
SrF_2	Yb^{3+}, Er^{3+}	1.1	0.3	25-110	$^2H_{11/2}$→$^4I_{15/2}$/ $^4S_{3/2}$→$^4I_{15/2}$	525/545	7
β-$NaYF_4$/SiO_2 *core/shell*	Yb^{3+}, Er^{3+}	≈1	1.1	27-627	$^2H_{11/2}$→$^4I_{15/2}$/ $^4S_{3/2}$→$^4I_{15/2}$	520/545	8
LaF_3:Yb,Er/ LaF_3:Yb,Tm *core/shell*	Yb^{3+}, Er^{3+}, Tm^{3+}	2.1<br>2.5	≈0.7<br>≈0.6	20-50	(Yb^{3+}) $^2F_{7/2}$→$^2F_{5/2}$/ (Tm^{3+}) $^3H_5$→$^3H_6$<br>(Yb^{3+}) $^2F_{7/2}$→$^2F_{5/2}$/ (Er^{3+}) $^4I_{13/2}$→$^4I_{15/2}$	1000/ 1230<br>1000/ 1550	9
SrF_2:Yb,Tm/ Y/Yb,Er,Nd/ Nd *core/shell*	Yb^{3+}, Nd^{3+}, Er^{3+}, Tm^{3+}	1.45	1.7	20-60	(Yb^{3+}) $^2F_{5/2}$→$^2F_{7/2}$/ (Nd^{3+}) $^4F_{3/2}$→$^4I_{11/2}$	980/1060	10

References:

(1) Zheng, T.; Runowski, M.; Martín, I. R.; Lis, S.; Vega, M.; Llanos, J. Nonlinear Optical Thermometry—A Novel Temperature Sensing Strategy via Second Harmonic Generation (SHG) and Upconversion Luminescence in BaTiO 3 :Ho 3+ ,Yb 3+ Perovskite. *Adv. Opt. Mater.* **2021**, *9* (12), 2100386. https://doi.org/10.1002/adom.202100386.

(2) Zheng, T.; Runowski, M.; Woźny, P.; Barszcz, B.; Lis, S.; Vega, M.; Llanos, J.; Soler-Carracedo, K.; Martín, I. R. Boltzmann vs. Non-Boltzmann (Non-Linear) Thermometry - Yb3+-Er3+ Activated Dual-Mode Thermometer and Phase Transition Sensor via Second Harmonic Generation. *J. Alloys Compd.* **2022**, *906*, 164329. https://doi.org/10.1016/j.jallcom.2022.164329.

(3) Runowski, M.; Goderski, S.; Przybylska, D.; Grzyb, T.; Lis, S.; Martín, I. R. Sr 2 LuF 7 :Yb 3+ –Ho 3+

–Er 3+ Upconverting Nanoparticles as Luminescent Thermometers in the First, Second, and Third Biological Windows. *ACS Appl. Nano Mater.* **2020**, *3* (7), 6406–6415. https://doi.org/10.1021/acsanm.0c00839.

(4) Pudovkin, M. S.; Morozov, O. A.; Pavlov, V. V; Korableva, S. L.; Lukinova, E. V; Osin, Y. N.; Evtugyn, V. G.; Safiullin, R. A.; Semashko, V. V. Physical Background for Luminescence Thermometry Sensors Based on Pr 3+ :LaF 3 Crystalline Particles. *J. Nanomater.* **2017**, *2017*, 1–9. https://doi.org/10.1155/2017/3108586.

(5) Rocha, U.; Jacinto Da Silva, C.; Ferreira Silva, W.; Guedes, I.; Benayas, A.; Martínez Maestro, L.; Acosta Elias, M.; Bovero, E.; Van Veggel, F. C. J. M.; García Solé, J. A.; et al. Subtissue Thermal Sensing Based on Neodymium-Doped LaF3nanoparticles. *ACS Nano* **2013**, *7* (2), 1188–1199. https://doi.org/10.1021/nn304373q.

(6) Runowski, M.; Stopikowska, N.; Szeremeta, D.; Goderski, S.; Skwierczyńska, M.; Lis, S. Upconverting Lanthanide Fluoride Core@Shell Nanorods for Luminescent Thermometry in the First and Second Biological Windows: β-NaYF 4 :Yb 3+ – Er 3+ @SiO 2 Temperature Sensor. *ACS Appl. Mater. Interfaces* **2019**, *11* (14), 13389–13396. https://doi.org/10.1021/acsami.9b00445.

(7) Balabhadra, S.; Debasu, M. L.; Brites, C. D. S.; Ferreira, R. A. S.; Carlos, L. D. Upconverting Nanoparticles Working As Primary Thermometers In Different Media. *J. Phys. Chem. C* **2017**, *121* (25), 13962–13968. https://doi.org/10.1021/acs.jpcc.7b04827.

(8) Geitenbeek, R. G.; Prins, P. T.; Albrecht, W.; Van Blaaderen, A.; Weckhuysen, B. M.; Meijerink, A. NaYF4:Er3+,Yb3+/SiO2Core/Shell Upconverting Nanocrystals for Luminescence Thermometry up to 900 K. *J. Phys. Chem. C* **2017**, *121* (6), 3503–3510. https://doi.org/10.1021/acs.jpcc.6b10279.

(9) Ximendes, E. C.; Rocha, U.; Sales, T. O.; Fernández, N.; Sanz-Rodríguez, F.; Martín, I. R.; Jacinto, C.; Jaque, D. In Vivo Subcutaneous Thermal Video Recording by Supersensitive Infrared Nanothermometers. *Adv. Funct. Mater.* **2017**, *27* (38), 1702249. https://doi.org/10.1002/adfm.201702249.

(10) Cortelletti, P.; Skripka, A.; Facciotti, C.; Pedroni, M.; Caputo, G.; Pinna, N.; Quintanilla, M.; Benayas, A.; Vetrone, F.; Speghini, A. Tuning the Sensitivity of Lanthanide-Activated NIR Nanothermometers in the Biological Windows. *Nanoscale* **2018**, *10* (5), 2568–2576. https://doi.org/10.1039/C7NR06141B.
